# Preclinical evaluation of the cardiac toxicity of HMR-1826, a novel prodrug of doxorubicin

**DOI:** 10.1038/sj.bjc.6690646

**Published:** 1999-09

**Authors:** D Platel, S Bonoron-Adèle, R K Dix, J Robert

**Affiliations:** Institut Bergonié, 180 rue de Saint-Genes, Bordeaux-Cedex, 33076, France; INSERM U441, Avenue du Haut-Lévêque, Pessac, 33600, France; Hoescht Marion Roussell, Bridgewater, NJ, USA

**Keywords:** cardiotoxicity, anthracyclines, prodrug therapy

## Abstract

Cardiotoxicity represents the major side-effect limiting the clinical use of anthracyclines, especially doxorubicin, in cancer chemotherapy. The use of non-toxic prodrugs, or of liposome-encapsulated drugs, allows a better targeting of the tumours and may, therefore, improve the tolerance to the treatment. Using the model of isolated perfused rat heart, we have evaluated the cardiotoxicity of a novel prodrug of doxorubicin, HMR-1826, which consists of the association of doxorubicin to glucuronic acid. We have compared the cardiac effects (developed pressure, contractility and relaxation of the left ventricle) induced by HMR-1826 to those induced by doxorubicin and Doxil, a liposomal form of doxorubicin. HMR-1826 was administered intravenously every other day for 11 days at doses of 50–200 mg kg^−1^ per injection while doxorubicin was administered according to the same protocol at doses of 1–3 mg kg^−1^ per injection. Doxorubicin strongly decreased the cardiac functional parameters at the doses of 2.5 and 3 mg kg^−1^ per injection. Doxil (3 mg kg^−1^) and HMR-1826 (50–150 mg kg^−1^) were largely devoid of cardiotoxicity. HMR-1826 only induced significant alterations of the cardiac function at the highest dose used (200 mg kg^−1^ per injection). These alterations were much lower than those of doxorubicin at 2.5 mg kg^−1^ per injection, despite similar general toxicity symptoms (weight loss, nose bleeding and diarrhoea) at these respective doses. Thus, HMR-1826 appeared about 100-fold less cardiotoxic than doxorubicin. © 1999 Cancer Research Campaign


British Journal of Cancer (1999) 81(1), 24–27
© 1999 Cancer Research Campaign
Article no. bjoc.1999.0646

### Preclinical evaluation of the cardiac toxicity of HMR-1826, a novel prodrug of doxorubicin

**D Platel[1], S Bonoron-Adèle[2], RK Dix[3] and J Robert[1]**

[1]Institut Bergonié, 180 rue de Saint-Genes 33076 Bordeaux-Cedex, France; [2]INSERM U441, Avenue du Haut-Lévêque 33600 Pessac, France; [3]Hoescht Marion Roussell, Bridgewater, NJ, USA

**Summary** Cardiotoxicity represents the major side-effect limiting the clinical use of anthracyclines, especially doxorubicin, in cancer chemotherapy. The use of non-toxic prodrugs, or of liposome-encapsulated drugs, allows a better targeting of the tumours and may, therefore, improve the tolerance to the treatment. Using the model of isolated perfused rat heart, we have evaluated the cardiotoxicity of a novel prodrug of doxorubicin, HMR-1826, which consists of the association of doxorubicin to glucuronic acid. We have compared the cardiac effects (developed pressure, contractility and relaxation of the left ventricle) induced by HMR-1826 to those induced by doxorubicin and Doxil, a liposomal form of doxorubicin. HMR-1826 was administered intravenously every other day for 11 days at doses of 50–200 mg $kg^{-1}$ per injection while doxorubicin was administered according to the same protocol at doses of 1–3 mg $kg^{-1}$ per injection. Doxorubicin strongly decreased the cardiac functional parameters at the doses of 2.5 and 3 mg $kg^{-1}$ per injection. Doxil (3 mg $kg^{-1}$) and HMR-1826 (50–150 mg $kg^{-1}$) were largely devoid of cardiotoxicity. HMR-1826 only induced significant alterations of the cardiac function at the highest dose used (200 mg $kg^{-1}$ per injection). These alterations were much lower than those of doxorubicin at 2.5 mg $kg^{-1}$ per injection, despite similar general toxicity symptoms (weight loss, nose bleeding and diarrhoea) at these respective doses. Thus, HMR-1826 appeared about 100-fold less cardiotoxic than doxorubicin.

**Keywords:** cardiotoxicity; anthracyclines; prodrug therapy

HMR-1826 is a novel prodrug of doxorubicin corresponding to an original concept for prodrug activation (Muerdter et al, 1997) that had already been considered for alkylating agents (Connors and Whisson, 1966). It consists of the standard anticancer drug doxorubicin, conjugated to glucuronic acid via a spacer (Figure 1). This relatively non-toxic compound is selectively activated in necrotic areas of tumours due to the liberation of lysosomal β-glucuronidase from acute and chronic inflammatory cells in disintegrating tumour tissue (Bosslet et al, 1998). There is good evidence that HMR-1826 generates superior therapeutic activity in several human tumour xenograft models over standard chemotherapy with doxorubicin (Bosslet et al, 1998).

One of the most important problems that has arisen during the widespread use of doxorubicin in cancer chemotherapy is related to its cardiotoxicity. This cardiotoxicity limits the cumulative dose of doxorubicin that can be administered to a maximum of 500–550 mg $m^{-2}$ (Von Hoff et al, 1979), a dose above which the risk of congestive heart failure increases in unacceptable proportions. This cumulative dose corresponds to about ten courses of treatment and prevents the drug from reaching its optimal activity in many patients, especially breast cancer patients (Mouridsen, 1992).

There has been intensive research in several pharmaceutical firms to develop either new anthracyclines with significantly less cardiotoxicity (such as epirubicin), or cardioprotectors able to decrease the cardiotoxicity of doxorubicin (such as dexrazoxane). Another approach has concentrated on developing drug-targeting systems able to increase doxorubicin availability in tumours while decreasing its systemic availability, in particular accumulation in cardiac tissue. Liposome-encapsulated doxorubicin (Working and Dayan, 1996), polymer-bound doxorubicin (Duncan et al, 1998) and prodrug monotherapy represent important achievements in this field, but the cardiotoxicity of these new formulations has not been studied in detail to date.

We have recently developed a functional ex vivo model for the rapid evaluation of the cardiotoxicity of anthracyclines, and this model was shown to correctly predict for the comparative cardiotoxicity of the various molecules presently marketed including the known activity of dexrazoxane as a cardioprotector

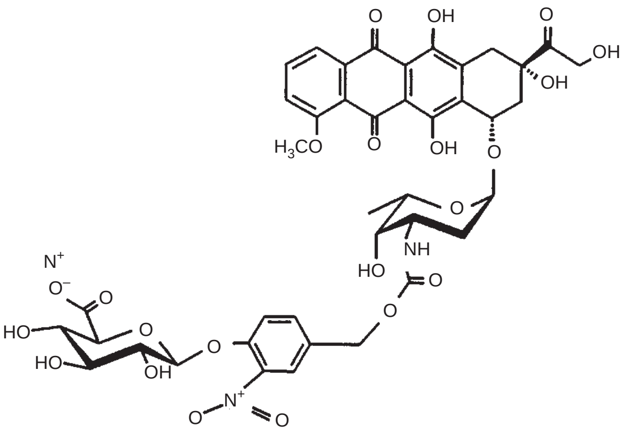

**Figure 1** Chemical structure of HMR-1826 (*N*-(4-β-glucuronosyl-3-nitrobenzyloxycarbonyl-doxorubicin)

*Received 5 October 1998*
*Revised 10 March 1999*
*Accepted 12 March 1999*

*Correspondence to:* J Robert

(Pouna et al, 1996). This model consists in treating rats every two days for 11 days with anthracyclines, at the dose of 3 mg $kg^{-1}$ per day (18 mg $kg^{-1}$ cumulative dose), intraperitonealy (i.p.). The myocardial functional performances are studied on the 12th day by perfusion of the isolated hearts and measure of the pressure developed in the left ventricle, according to Langendorff (Lorell et al, 1986).

We have evaluated the cardiotoxicity of HMR-1826 in this ex vivo model, when administered intravenously (i.v.) every other day for 11 days at doses of 50–200 mg $kg^{-1}$ per injection. We show here that this prodrug has a very low cardiotoxicity, which appeared only at the highest dose administered, while doxorubicin already exerts a significant cardiotoxicity at the dose of 2.5 mg $kg^{-1}$ per injection. Liposomal doxorubicin (Doxil), at 3 mg $kg^{-1}$ per injection, was also devoid of cardiotoxicity. These results warrant the introduction and evaluation of this new anthracycline prodrug in clinical trials.

### MATERIALS AND METHODS

### Drugs and chemicals

HMR-1826 was provided by Hoechst Marion Roussel (Marburg, Germany) as lyophilized powder which was reconstituted extemporaneously in 5% mannitol at the concentration of 25 mg $ml^{-1}$, and filtered on 0.22 µm filters. Doxorubicin was provided by Pharmacia & Upjohn (Saint-Quentin-en-Yvelines, France) as the clinical formulation, which was reconstituted in 0.9% sodium chloride (NaCl) at the concentration of 1 mg $ml^{-1}$, divided into aliquots and kept frozen until use. Liposomal doxorubicin (Doxil®, Caelyx®) was provided by Essex Pharma (München, Germany).

All other chemicals and solvents were of the highest grade commercially available. Special care was taken concerning the water used for the perfusion medium; we used sterile apyrogenic water specially prepared for parenteral injections in humans.

### Experimental animals

All animal experiments described in this report were done in accordance with the guidelines of Institut National de la Santé et de la Recherche Médicale. Male Sprague-Dawley rats aged 10–12 weeks and weighing 300–350 g were obtained from CERJ, Le Genest-Saint-Isle, France. All experiments included controls receiving either 0.9% NaCl (control group for doxorubicin and Doxil) or 5% mannitol (control group for HMR-1826). The drugs (doxorubicin at 1, 2, 2.5 or 3 mg $kg^{-1}$ per injection, Doxil at 3 mg $kg^{-1}$ per injection and HMR-1826 at 50, 100, 150 or 200 mg $kg^{-1}$ per injection) were administered i.v. via the tail vein on days 1, 3, 5, 7, 9 and 11 after weighing of the rats and assessment for possible general toxicity symptoms. On the 12th day the rats were killed, their hearts were removed and perfused, cardiac functional parameters were monitored as described below, and the hearts were weighed at the end of the experiment. The number of rats in each experimental group varied between 8 and 12 in all cases, except for the rats receiving HMR-1826 at doses of 50, 150 and 200 mg $kg^{-1}$ per injection, which were four or five in each group.

### Perfusion of isolated rat hearts

Rats were heparinized i.p. (500 IU per 100 g body weight) and anaesthetized with diethylether. The heart was quickly excised and soaked in Krebs–Henseleit solution at 4°C. Coronary perfusion was initiated through a short cannula in the aortic root and maintained at a constant pressure of 90 mmHg in a non-recirculating way by the Langendorff technique as described by Lorell et al (1986). Perfusion pressure was measured by a P23Db transducer (Bentley Trantec) connected to the aortic infusion cannula. The heart was electrically paced at a rate of 300 beats per min (5 Hz) through stimulator-activated stainless steel electrodes placed on the heart. A latex balloon attached to one end of a polyethylene catheter was placed in the left ventricle through the mitral valve. The catheter was filled with water and the other end was linked to an electronic amplifier (Thomson Medical) via a second P23Db transducer.

The coronary perfusion pressure and the left ventricular pressure were recorded on a computer that allowed continuous monitoring of heart rate, left ventricular systolic pressure (LVSP), left ventricular end-diastolic pressure (LVEDP), left ventricular developed pressure (LVDP = LVSP – LVEDP) and the maximal and minimal first derivatives of LVDP as a function of time ($LV(dP/dt)_{max}$ and $LV(dP/dt)_{min}$) respectively. The perfusate consisted in a modified Krebs–Henseleit buffer, pH 7.4, containing NaCl (118 mM), potassium chloride (4.7 mM), magnesium sulphate (1.2 mM), $KH_2PO_4$ (1.2 mM), sodium hydrogencarbonate (25 mM), glucose (11 mM), calcium chloride (0.95 mM) and insulin (10 IU $l^{-1}$). It was continuously bubbled with a mixture of 95% oxygen 5% carbon dioxide and maintained at 37°C. The latex balloon inserted in the left ventricle was periodically dilated with distilled water in order to produce a LVEDP of 5–6 mmHg. After 30- to 45-min stabilization, necessary to reach the maximal functional cardiac values, the above parameters were recorded.

### Anthracycline accumulation

Samples were obtained from the hearts of three rats treated according to the same protocol with doxorubicin (1 mg $kg^{-1}$ per injection) and of five rats treated with HMR-1826 (100 mg $kg^{-1}$ per injection) for the measure of cardiac accumulation of doxorubicin. These samples were homogenized in physiological saline (2 ml for 100 mg tissue) with a tissue homogenizer (Ultra-Turrax). After addition of an adequate amount of internal standard (daunorubicin) and of 0.5 ml borate buffer (50 mM, pH 9.8) to 0.5 ml of the homogenate, anthracyclines and metabolites were extracted with 9 ml of chloroform–methanol 4/1 (v/v), according to Baurain et al (1979). After mixing and centrifuging (10 min at 3000 ***g***), the solvent layer was recovered, evaporated to dryness and reconstituted with 200 µl methanol. Calibration curves were obtained after incubating heart homogenates with doxorubicin in vitro for 15 min at room temperature. A good linearity was obtained from 0.015 to 1.5 nmol $g^{-1}$ tissue. Chromatography was performed on a Radial-Pak C18 column (Waters Associates, Saint-Quentin-en-Yvelines, France) inserted in a compression device. The solvent was a mixture of ammonium formate buffer (60 mM, pH 4.0) and acetonitrile (68/32) delivered at 2 ml $min^{-1}$. Detection was achieved with a laser-induced fluorescence recorder (Zeta Technology, Toulouse, France) with excitation and emission wavelengths set at 488 and 550 nm respectively. Retention times and peak areas were recorded with a micro computer using the PC1000 software (Thermo Quest, Les Ulis, France).

*© 1999 Cancer Research Campaign*

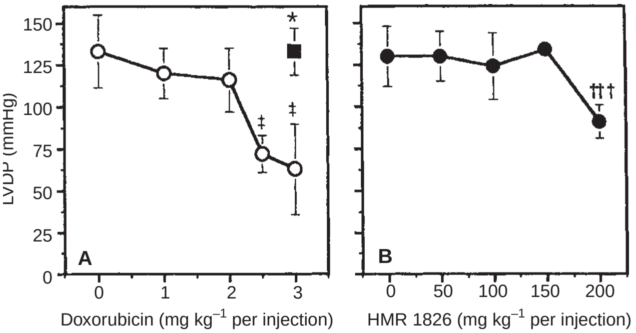

**Figure 2** Effect of drug treatments on the left ventricular developed pressure. (**A**) Doxorubicin (○) and Doxil (■). (**B**) HMR-1826. Student's *t*-test: treated vs control rats: †††$P < 0.001$; ‡$P < 0.0001$; Doxil at 3 mg kg$^{-1}$ vs doxorubicin at 3 mg kg$^{-1}$: *$P < 0.0001$

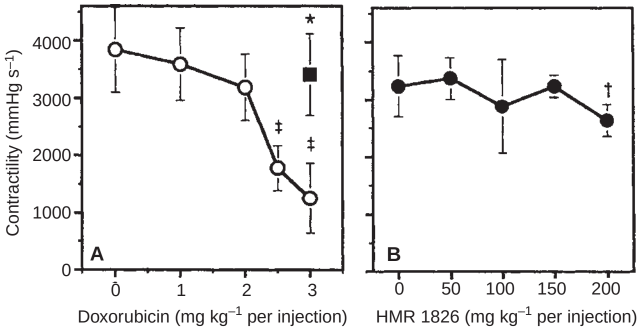

**Figure 3** Effect of drug treatments on the left ventricular contractility. (**A**) Doxorubicin (○) and Doxil (■). (**B**) HMR-1826. Student's *t*-test: treated vs control rats: †$P < 0.05$; ‡$P < 0.0001$; Doxil at 3 mg kg$^{-1}$ vs doxorubicin at 3 mg kg$^{-1}$: *$P < 0.0001$

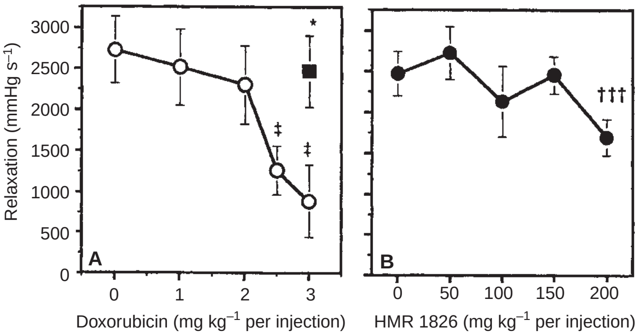

**Figure 4** Effect of drug treatments on the left ventricular relaxation. (**A**) Doxorubicin (○) and Doxil (■). (**B**) HMR-1826. Student's *t*-test: treated vs control rats: †††$P < 0.001$; ‡$P < 0.0001$; Doxil at 3 mg kg$^{-1}$ vs doxorubicin at 3 mg kg$^{-1}$: *$P < 0.0001$

### Statistical analysis of the data

Statistical comparisons between untreated and treated groups were made by Student's *t*-test after ANOVA assumption of the validity of *t*-test; all data in the tables are expressed as mean value ± s.d. Statistical significance was determined as a *P*-value below 0.05.

### RESULTS AND DISCUSSION

The general toxicity of the treatment with doxorubicin and HMR-1826 could be evaluated by the decrease in body weight. There was a parallel decrease in body weight induced by the two drugs, with a shift between the two curves: a similar loss was obtained with doxorubicin at the dose of 2.5 mg kg$^{-1}$ per injection and HMR-1826 at 200 mg kg$^{-1}$ per injection. The equivalent dose ratio was therefore around 80 for this parameter. When considering other general toxicity symptoms, it appeared that doxorubicin provided nose bleeding at the dose of 2 mg kg$^{-1}$ per injection and beyond, and diarrhoea from the dose of 2.5 mg kg$^{-1}$ per injection. HMR-1826 induced nose bleeding at 150 mg kg$^{-1}$ per injection and both nose bleeding and diarrhoea at 200 mg kg$^{-1}$ per injection. For these general toxicity symptoms, the equivalent dose ratio HMR-1826/doxorubicin also appeared to be around 75–80. Doxil at 3 mg kg$^{-1}$ per injection provided a similar weight loss and similar general toxicity symptoms as doxorubicin at 2 mg kg$^{-1}$ per injection.

The cardiac toxicity of the treatments could be evaluated by three parameters: the left ventricular developed pressure and the maximal and minimal first derivatives of the left ventricular pressure as a function of time, which evaluate the contractility and the relaxation properties of the left ventricle respectively. Figures 2–4 represent these parameters as a function of the dose administered. Doxorubicin induced significant alterations of the three parameters from the dose of 2.5 mg kg$^{-1}$ per injection. At this dose level, the decrease in LVDP, $LV(dP/dt)_{max}$ and $LV(dP/dt)_{min}$ were of about 50% of the control values. At 3 mg kg$^{-1}$ per injection, the alterations were extremely important and reached 70% of the control values. In comparison, Doxil induced no significant alterations of cardiac functional parameters at the dose of 3 mg kg$^{-1}$ per injection, and HMR-1826 induced significant alterations only at the highest dose tested. However at this dose (200 mg kg$^{-1}$ per injection), the decrease in the functional parameters reached no more than 30%, still significantly less than with the dose of doxorubicin of 2.5 mg kg$^{-1}$ per injection. For cardiotoxicity, the equivalent dose ratio HMR-1826/doxorubin appears, therefore, to be higher than the equivalent dose ratio for general toxicity. This means that, at the dose providing the same general toxicity, HMR-1826 is significantly less cardiotoxic than doxorubicin. Furthermore, since a dose of 200 mg kg$^{-1}$ administered once every 3 weeks has been shown to significantly inhibit tumour xenografts (personal communication), this finding of significantly less cardiotoxicity on an every other day schedule may have clinical implications for a less frequent schedule.

Doxorubicin accumulation was measured in the heart of rats treated with the same protocol at doses of doxorubicin and Doxil of 1 mg kg$^{-1}$ per injection, and of HMR-1826 of 100 mg kg$^{-1}$ per injection. This accumulation on the 12th day amounted to 4.4 ± 0.8 nmoles g$^{-1}$ tissue for doxorubicin, which was 30 times higher than the accumulation obtained after Doxil treatment (0.14 ± 0.03 nmoles g$^{-1}$ tissue, $P < 0.01$ vs doxorubicin) and threefold higher than following HMR-1826 administration (1.5 ± 0.2 nmoles g$^{-1}$ tissue, $P < 0.01$ vs doxorubicin and $P < 0.0001$ vs Doxil). Since heart doxorubicin likely originates from circulating free doxorubicin released from the vehicle, this would mean that only 0.3% of the glucuronide injected generates free doxorubicin in the circulation. Doxorubicinol concentration never exceeded 10% of doxorubicin concentration in all three groups and did not significantly differ between groups. These preliminary data suggest that the lower cardiotoxicity of HMR-1826 as compared to doxorubicin may be due to its reduced accumulation in the heart.

 © 1999 Cancer Research Campaign

In a comparison of the anti-tumour activities of doxorubicin and HMR-1826, Bosslet et al (1998) showed a significantly higher inhibition of tumour growth in most of the 20 human xenograft models explored, for dose ratios HMR-1826/doxorubicin between 25 and 133. It appears, therefore, that this new drug formulation widens the therapeutic window of doxorubicin.

### ACKNOWLEDGEMENTS

This work was made feasible thanks to a grant from Hoechst Marion Roussel. We are grateful to Mrs L Tariosse and Mr G Gouverneur for technical assistance.

### REFERENCES

Baurain R, Deprez-De Campaneere D and Trouet A (1979) Rapid determination of doxorubicin and its fluorescent metabolites by high pressure liquid chromatography. *Anal Biochem* **94**: 112–116

Bosslet K, Straub R, Blumrich M, Czech J, Gerken M, Sperker B, Kroemer HK, Gesson JP, Koch M and Monneret C (1998) Elucidation of the mechanism enabling tumor selective prodrug monotherapy. *Cancer Res* **58**: 1195–1201

Connors TA and Whisson ME (1966) Cure of mice bearing advanced plasma cell tumours with aniline mustard: the relationship between glucuronidase activity and tumour sensitivity. *Nature* **210**: 866–867

Duncan R, Coatsworth JK and Burtles S (1998) Preclinical toxicology of a novel polymeric antitumour agent: HPMA copolymer-doxorubicin (PK1). *Human Exp Toxicol* **17**: 93–104

Florent JC, Gaudel G, Mitaku S, Monneret C, Gesson JP, Jacquesy JC, Mondon J, Martine C, Renoux B, Adrianomenjanahary S, Michel S, Koch M, Tillequin F, Gerken M, Czech J, Straub R and Bosslet K (1998) Prodrugs of anthracyclines for use in antibody-directed enzyme prodrug therapy. *J Med Chem* **41**: 3572–3581

Lorell BH, Wexler LF, Momomura S, Weinberg E and Apstein CS (1986) The influence of pressure overload left ventricular hypertrophy on diastolic properties during hypoxia in isovolumically contracting rat hearts. *Circ Res* **58**: 653–663

Mouridsen HT (1992) Systemic therapy for advanced breast cancer. *Drugs* **44**: 17–28

Muerdter TE, Sperker B, Kivistö KT, McClellan M, Fritz P, Friedel G, Linder A, Bosslet K, Toomes H, Dierkesmann R and Kroemer HK (1997) Enhanced uptake of doxorubicin into bronchial carcinoma: β-glucuronidase mediates release of doxorubicin from a glucuronide prodrug. *Cancer Res* **57**: 2440–2445

Pouna P, Bonoron-Adèle S, Gouverneur G, Tariosse L, Besse P and Robert J (1996) Development of the model of rat isolated perfused heart for the evaluation of anthracycline cardiotoxicity and its circumvention. *Br J Pharmacol* **117**: 1593–1599

Von Hoff DD, Layard MW, Basa P, Davis HL, Von Hoff AL, Rozencweig M and Muggia FM (1979) Risk factors for doxorubicin-induced congestive heart failure. *Ann Intern Med* **91**: 710–717

Working PK and Dayan AD (1996) Pharmacological-toxicological expert report: Caelyx™ (Stealth® liposomal doxorubicin HCl). *Human Exp Toxicol* **15**: 752–785

*© 1999 Cancer Research Campaign* 
